# Non‐invasive prenatal testing in the management of twin pregnancies

**DOI:** 10.1002/pd.5989

**Published:** 2021-06-25

**Authors:** Peter Benn, Andrei Rebarber

**Affiliations:** ^1^ Department of Genetics and Genome Sciences UConn Health Farmington Connecticut USA; ^2^ Department of Obstetrics, Gynecology, and Reproductive Sciences Icahn School of Medicine at Mount Sinai New York New York USA; ^3^ Division of Maternal Fetal Medicine Englewood Hospital Englewood New Jersey USA

## Abstract

Twin pregnancies are common and associated with pregnancy complications and adverse outcomes. Prenatal clinical management is intensive and has been hampered by inferior screening and less acceptable invasive testing. For aneuploidy screening, meta‐analyses show that non‐invasive prenatal testing (NIPT) through analysis of cell‐free DNA (cf‐DNA) is superior to serum and ultrasound‐based tests. The positive predictive value for NIPT is driven strongly by the discriminatory power of the assay and only secondarily by the prior risk. Uncertainties in a priori risks for aneuploidies in twin pregnancies are therefore of lesser importance with NIPT. Additional information on zygosity can be obtained using NIPT. Establishing zygosity can be helpful when chorionicity was not reliably established early in pregnancy or where the there is a concern for one versus two affected fetuses. In dizygotic twin pregnancies, individual fetal fractions can be measured to ensure that both values are satisfactory. Vanishing twins can be identified by NIPT. Although clinical utility of routinely detecting vanishing twins has not yet been demonstrated, there are individual cases where cf‐DNA analysis could be helpful in explaining unusual clinical or laboratory observations. We conclude that cf‐DNA analysis and ultrasound have synergistic roles in the management of multiple gestational pregnancies.

## Key Points

What's already known about this topic?

- In singleton pregnancies, non‐invasive prenatal testing (NIPT) for fetal aneuploidy is more effective than conventional serum and ultrasound‐based screening tests.
- NIPT is more complex in dizygotic twin pregnancies due to the presence of two fetal genotypes.
- Overall fetal fraction is higher in twin pregnancies but the individual contribution for each fetus is lower.

What does this study add?

- A review of cell‐free DNA testing in twin pregnancies.
- Individual fetal fractions in dizygotic twin pregnancies can be measured.
- Zygosity can be established using NIPT and this can be particularly useful when there are concerns about chorionicity or determining whether one versus two fetuses are affected.
- Vanishing twins can be detected through NIPT and this testing could be considered for some apparently singleton pregnancies with complications.

## 1 INTRODUCTION

In the United States, in 2018, approximately one in 33 births was the product of a twin gestation.^1^ Due to the increased use of assisted reproductive technologies and increasing maternal age at conception, rates of twins have increased in recent decades. The rate of twinning is also very high for many other countries.^2^

Multifetal pregnancies are at increased risk for a broad range of pregnancy complications and adverse outcomes.^3^, ^4^ Although only a minority of twin pregnancies are monochorionic, they account for a high portion of the perinatal morbidity and mortality. Maternal age‐specific risks for fetal trisomy 21 are not increased,^5^ but traditional screening tests have been less effective for women with twin pregnancies.^6^, ^7^ Women with twin pregnancies may have been more reluctant to undergo invasive testing (chorionic villus biopsy [CVS] or amniocentesis) as a result of studies that have indicated increased procedure‐related risks of pregnancy loss.^8^, ^9^, ^10^ Multiple pregnancies are therefore common, associated with a complex set of adverse outcomes, and management has been hampered by late gestational age and less acceptable screening and diagnostic options. Management of twin pregnancies has been associated with higher healthcare costs.^11^, ^12^

For women with twin pregnancies, the introduction of non‐invasive prenatal testing (NIPT) through analysis of cell‐free DNA (cf‐DNA) in maternal circulation offers significantly improved aneuploidy screening, reduced need for invasive testing, and is available from late in the first trimester. Additional information on zygosity can also be obtained. NIPT in twin pregnancies is now endorsed by the International Society for Prenatal Diagnosis.^2^ The American College of Obstetrics and Gynecology also notes that the testing can be offered.^13^ This testing is therefore recognized as an option in the advancement of clinical management and care of twin pregnancies.

In this paper, we review the application of this technology in multiple pregnancies. We begin by discussing the basic biology of twin pregnancies and review the data on cf‐DNA present in twin pregnancies. We then explain in detail how cf‐DNA analysis is improving clinical management of twin pregnancies.

## 2 ZYGOSITY AND CHORIONICITY

Approximately 60%–70% of twin pregnancies are dizygotic and 20%–30% monozygotic, depending on the maternal age, use of fertility treatments, and race/ethnicity of the population. Dizygotic (“non‐identical”) twins are the product of two egg fertilizations and these pregnancies will generally have dichorionic diamniotic extra‐fetal tissues.^14^ The existence of monochorionicity for dizygotic twins has traditionally been viewed as rare although more recent studies suggest this may be more frequently encountered, especially when there has been assisted reproduction.^15^ Monozygotic (“identical”) twins are the consequence of an early splitting of the cells derived from a single fertilized egg and the presentation of the extra‐embryonic tissues will be dependent on the timing of the split. Very early separation (morula stage) will result in dichorionic diamniotic tissues (i.e., the same as that seen in a dizygotic twin pregnancy). Separation at the hatching stage results in monochorionic diamniotic tissues. Later separation results in monochorionic monoamniotic tissues. Approximately 75% of monozygotic twins are monochorionic and 25% are dichorionic. There are also very rare examples of twins in which only the chromosome set from one parent is shared.^16^, ^17^

Although all twin pregnancies are considered to be high‐risk for almost all obstetrical complications in pregnancy (except post‐date pregnancies and macrosomia), the primary associated risk factor for a poor pregnancy outcome in twin pregnancies is the chorionicity.^18^ Monochorionic twins are uniquely at risk for twin‐to‐twin transfusion syndrome (TTTS), twin anemia‐polycythemia syndrome and twin reversed arterial perfusion. Chorionicity can be established by ultrasound examination in the first trimester. The characteristic “lambda” sign on ultrasound is considered to be indicative of monochorionicity and has generally been considered to be accurate provided the sonogram is carried out prior to 14 completed weeks gestational age.^19^ However, not all patients are able to obtain this early diagnostic ultrasound. For example, based on Center for Disease Control reports for 2016, in the United States, over 20% of women entered prenatal care in the second trimester or later and this proportion was even greater in marginalized and vulnerable populations.^20^ It is not always possible to determine chorionicity due to intrauterine crowding of the fetuses, reduced amniotic fluid, or maternal obesity.

Some studies have questioned the accuracy of ultrasound to assign chorionicity. In one large study that assumed placental pathology was accurate, the sensitivity of detecting monochorionicity was 89.8% and specificity 99.5%.^21^ In another study, for women receiving sonography prior to 20 weeks, 18 of 455 dichorionic twins (4.0%) and 17 of 90 monochorionic twins (19.0%) were mis‐classified with greater accuracy for sonograms performed prior to 14 weeks.^22^ Monochorionic twin pregnancies require early and repeated ultrasound exams and additional surveillance to identify early signs for complications. Specialized pregnancy counseling by a Maternal Fetal Medicine specialist is advised to explain the prenatal risks, management, and care. Incorrect assignment of monochorionicity can potentially entail unnecessary use of clinical resources and patient stress while under‐calling can result in additional adverse outcomes.^23^

## 3 CELL‐FREE DNA IN TWIN PREGNANCIES

**FIGURE 1:**
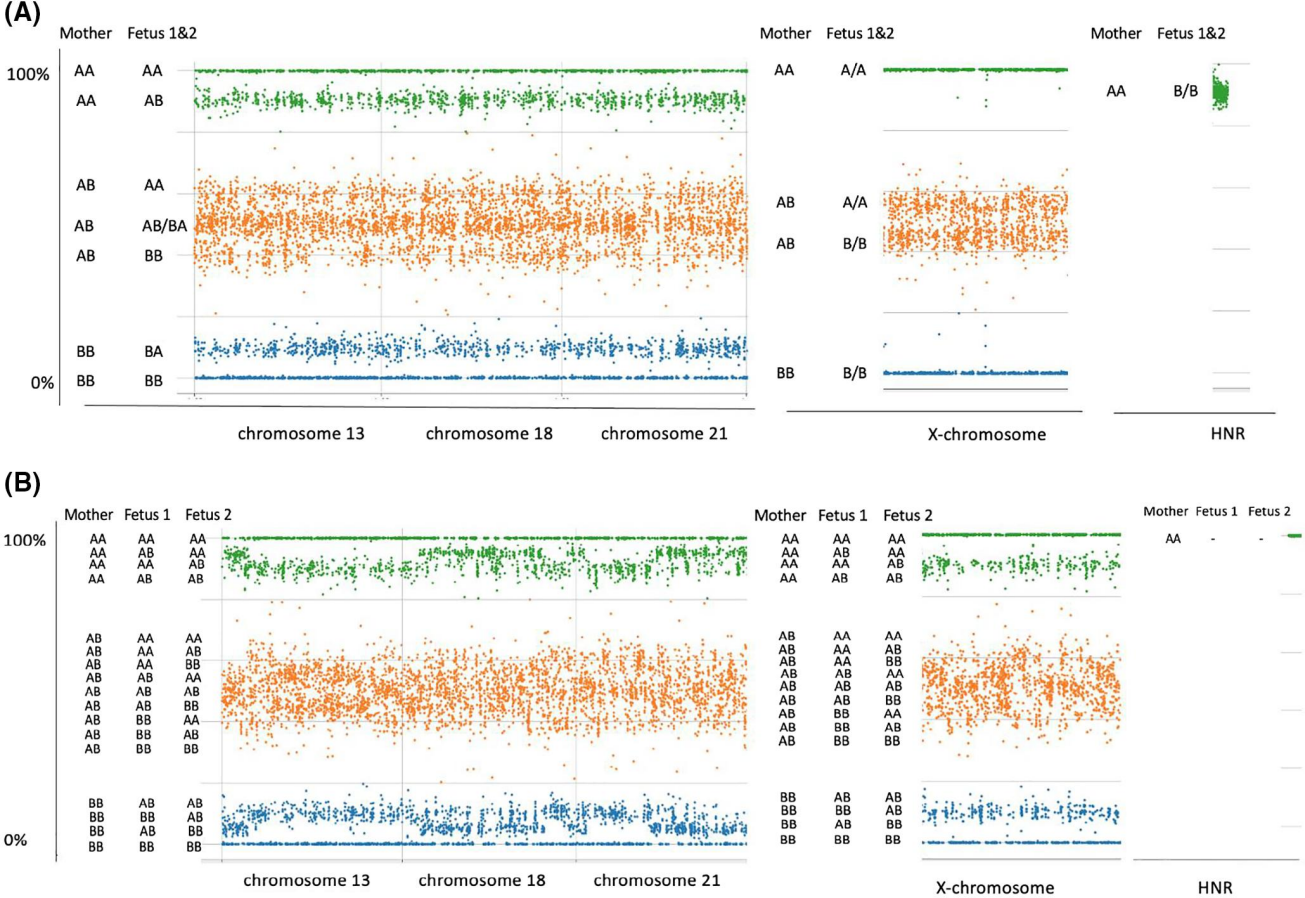
Heterozygosity plots for single nucleotide polymorphisms (SNPs) in euploid twin pregnancies. (A) monozygotic twins. (B) dizygotic twins. Heterozygosity plots are visual graphical representations of the alleles present in the maternal plasma. In these figures, each SNP type is denoted as either an “A” or “B” allele. The *x*‐axis, left to right, shows the alleles along chromosomes 13, 18, 21, X and a set of homologous non‐recombinant regions (HNR) from the X and Y chromosomes. The HNR SNPs are designated “A” when they map to the X‐chromosome and “B” when they map to the Y‐chromosome. The *Y*‐axis shows, for each locus present, the overall percentage of A type, that is, (A/[A + B])%. The *Y* axis also specifies the corresponding allele combinations for the mother and the fetuses. The data is shown in green if the maternal genotype is AA, blue BB, or orange AB. (A) Euploid male monozygotic twins. The patten is the same as that which would be present for a singleton pregnancy. The extent to which the lower green band departs from 100%, the two orange bands depart from 50%, and upper blue band departs from 0% is determined by the fetal fraction. Thus, all informative SNPs provide a measure of the fetal fraction. The SNP pattern for the X‐chromosome indicates that there are no paternally derived X‐chromosomes present in the cell‐free DNA (cf‐DNA), consistent with a male fetal sex. Moreover, the HNR SNPs indicate presence of Y‐chromosome alleles at a level consistent with both fetuses being male. (B) Euploid female dizygotic twins. There are additional allelic combinations, compared to the monozygotic pattern, that are attributable to the presence of a second paternally derived haplotype. Some of the paternally derived SNPs will be identical in the two fetuses and others will differ. Although band patterns appear to be more diffuse and overlapping in the graphical representation, the allele contributions from each fetus can be computationally resolved (allowing for linkage and recombination) and the individual fetal fractions can be separately determined, similar to that for singleton pregnancies.^25^ The X‐chromosome pattern is similar to the autosomes when the sex of the fetuses is female. The HNR SNPs indicate no Y‐chromosome contribution. For SNP‐based NIPT in twins, initially evaluating zygosity is necessary in the assessment of the presence or absence of aneuploidy. Presence of aneuploidy or a specific microdeletion would be indicated by a larger change in the A/(A + B) ratios, compared to that seen for the disomic chromosome regions (not shown). Illustration constructed from material provided by Natera, Inc

Similar to singletons, women with multiple pregnancies have cf‐DNA derived from trophoblasts in their maternal circulation (commonly referred to as “fetal” DNA). As a biomarker, fetal cf‐DNA has an inherent theoretical advantage over conventional maternal serum‐based tests because it provides a direct reflection of conceptus genotype and it can be evaluated through exquisitely powerful molecular technologies such as PCR and sequencing. Analysis of single nucleotide polymorphisms (SNPs) in cf‐DNA allows distinction between maternal and fetal sequences. In dizygotic twin pregnancies, SNPs can also allow an assessment of the cf‐DNA from each separate fetus. This can be done as a separate analytic component of a counting‐based NIPT^24^ or integrated into the overall assessment of zygosity and aneuploidy, as described in the legend to Figure 1.

**FIGURE 2:**
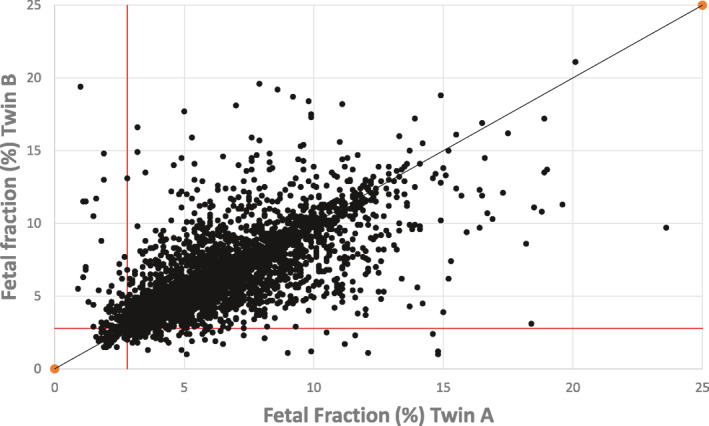
Scatterplot of the two fetal fractions in dizygotic twin pregnancies. Each fetal fraction (FF) in dizygotic pregnancies was randomly assigned as “Fetus A” or “Fetus B” and plotted. The plot illustrates that some pregnancies show large differences between the paired results (overall *R* ^2^ = 0.66). Points below the horizontal line or to the left of the vertical line have a least one fetal fraction below 2.8%, the cut‐off used for a reportable test. Data is based on 3161 dizygotic pregnancies previously reported by Hedriana et al.^29^

The fetal fraction of cfDNA in maternal plasma for women with twin pregnancies is higher, but less than two‐fold, than for women with singleton pregnancies.^26^, ^27^, ^28^, ^29^ For monozygotic pregnancies, this overall higher concentration and the fact that both fetuses almost always have the same genotype means that NIPT for genetic conditions will be equivalent, or better, than that for singletons.^30^ Conversely, for dizygotic twins, the individual cf‐DNA contributed by each fetus is generally lower than that for a singleton and usually only one fetus will be affected with an aneuploidy. Using SNP analyses, the cf‐DNA corresponding to each twin can be separately assessed and analyzed (in addition to distinguishing fetal from the maternal cf‐DNA). SNP analyses indicate that in twin pregnancies the individual cfDNA concentrations from each fetus are only moderately correlated with each other. One fetal fraction can be high and the other below the cut‐off for reliable testing (Figure 2). Concerns about the adequacy of the fetal fraction will be greatest when screening for conditions such as trisomy 18, trisomy 13 and digynic triploidy (when offered) where fetal fraction is known to be low due to the presence of less placental tissue.^31^, ^32^, ^33^, ^34^, ^35^, ^36^ Optimal screening that is inclusive of these conditions therefore involves measurement of both individual cf‐DNA fetal fractions. Dizygotic twin pregnancies provide an ideal opportunity to investigate individual fetal factors that might cause an aberrant fetal fraction concentration with the second twin as a control and with both twins subject to the identical maternal conditions.

## 4 USE OF CF‐DNA TO ASSESS ZYGOSITY

From the above discussion on the ability to detect different fetal genotypes it should be clear that allele specific SNPs or other polymorphic differences between fetuses can distinguish monozygotic and di‐zygotic twin pregnancies in the late first, second and third trimesters of pregnancy. A validation study for a SNP‐based zygosity test demonstrated 100% sensitivity and specificity in a series of 95 twin pregnancies.^30^ For zygosity assessment, no precise quantification of the relative amounts of each genotype is necessary; the presence of extra allele combinations in a dizygotic pregnancy is sufficient (Figure 1). The difference between monozygotic and dizygotic SNP patterns can be seen even at relatively low fetal fractions. One caveat is that triploidy can also contribute an extra set of alleles similar to that seen in dizygotic twins. However, that distinction can be made from the ultrasound findings. Zygosity can also be established through analyses of short tandem repeat and deletion/insertion DNA polymorphisms.^37^

Some examples of the clinical value of the application of zygosity testing are as follows:

1. Cases where chronicity evaluation by ultrasound was not carried out due to late gestational age at initiation of care or was otherwise not available, imaging was equivocal, or where subsequent clinical findings were suggestive of an incorrect chorionicity assignment. When dizygosity is established, the pregnancy is very likely to be dichorionic. If monozygosity is established, there is an approximately 75% chance of monochorionicity and 25% chance of dichorionicity. Zygosity testing is not a replacement for routine first trimester ultrasound evaluation of chorionicity.
2. Cases where there was fetal anatomic abnormality or growth restriction in only one of the two fetuses and there was a concern regarding both fetuses being affected by a variably expressed or variably penetrant disorder versus one affected and one unaffected. Zygosity testing can alter the risk that one of the fetuses is unaffected and this may affect patient choice and clinical management. For example, when monozygosity has been established, restricting CVS or amniocentesis to only the anatomically abnormal fetus might be preferred.
3. As an adjunct to other NIPT studies. For example, for pregnancies with aneuploidy positive results, monozygosity implies both are very likely to be affected versus dizygosity where, generally, only one will be affected. Similarly, in cases with suspected mosaicism, zygosity can help establish whether one or both fetuses are likely to be affected (cf‐DNA alone will not determine which of the two dizygotic twins is affected).
4. In cases with insufficient fetal cf‐DNA concentration for an aneuploidy result. If zygosity can be established, risk can be modified prior to offering an alternative screening or diagnostic test (see below).

## 5 USE OF CF‐DNA TO SCREEN FOR ANEUPLOIDY

In theory, since there are two separate conceptions in a dizygotic pregnancy, it would be expected that the maternal age‐specific per pregnancy rate for aneuploidy would be twice that for a singleton pregnancy and the rate for monozygotic twin pregnancies which are derived from a single egg would be similar to singletons.^38^ In practice, for trisomy 21, the observed prevalence of either type of affected pregnancy is lower than expected, presumably due to increased affected pregnancy loss.^39^, ^40^ The ratio of prevalence rates across maternal ages are not the same for monozygotic and dizygotic twins; the rate of dizygotic, but not monozygotic, twining is higher in older women and there are geographic differences in rates.^41^ Use of assisted reproductive technology further complicates estimates of the a priori aneuploidy risk in multiples because maternal age needs to be based on that for the egg (that may be from a donor) at the time of retrieval. Rates of other chromosome abnormalities in twin pregnancies have not been established from direct observations and estimates are based on their proportionate relationship to trisomy 21 in singleton pregnancies.^5^

Conventional combined serum and ultrasound‐based screening for aneuploidy has been shown to have a lower detection rate and/or higher false‐positive rate than that achievable in singletons.^6^ Maternal serum markers are unable to distinguish between monozygotic and dizygotic twins and since most affected pregnancies are dizygotic with one affected fetus, serum marker tests are expected to be less discriminatory than is the case for singleton pregnancies.^7^ Conventional screening may also be less effective because assisted reproductive techniques (ARTs) may affect first trimester serum markers such as PAPP‐A and free‐*β* HCG levels in a way that mimics Down syndrome.^42^ The serum correction factors used for ART pregnancies may be imprecise and therefore further compound the error in the analysis of twin gestations. Finally, the false‐positive rate of nuchal translucency screening for aneuploidy is higher in monochorionic than dichorionic twins because increased nuchal translucency can be an early manifestation of TTTS.^43^

The option of invasive testing is also more problematic in twin pregnancies. The risk of miscarriage has been reported to be higher in twin pregnancies compared to singleton pregnancies,^3^, ^10^ although the most recent data suggests that the excess risk may be less than originally reported.^9^ Women who have had difficulties becoming pregnant and who received assisted reproduction with multiple embryo transfer often do not want to expose their pregnancy to any additional risk. Complications of sampling are also present in multiple gestations, particularly for CVS compared to amniocentesis. These complications include sampling one fetus twice, cross contamination due to mixed sampling, and maternal cell contamination. Operator experience, as well as a high volume of tests performed at the center, can ameliorate the frequency of these potential complications but never completely excludes them. Therefore, there may be a greater reluctance by women with twin pregnancies to undergo an invasive test.

Uncertainties in prior risk, limitations in distinguishing monozygotic and dizygotic twins by imaging, and decreased acceptability of follow‐up invasive testing therefore limit the efficacy of conventional approaches to aneuploidy detection in twin pregnancies. The importance of a well‐defined estimate of the prior risk becomes much less important with cf‐DNA testing where the positive predictive value is driven much more strongly by the discriminatory power of the assay and only secondarily by the prior risk.^44^ There is therefore a strong expectation that the cf‐DNA based testing would be highly effective for twin pregnancies.

Robust estimates of sensitivity are difficult to establish because of the relative rarity of affected pregnancies. Combining two recent meta‐analyses,^2^, ^45^ the sensitivity for trisomy 21 was 101/102 (99.0%) and the specificity was 6/6611 (0.09%). These data included both monozygotic and dizygotic twins, various approaches to testing (mostly counting‐based NIPT), generally unspecified prior testing, various criteria for test referral and differences in the criteria for a sufficient fetal fraction. Overall, the observed performance for trisomy 21 screening in twin pregnancies has been comparable to that observed for singleton pregnancies, albeit with a higher proportion of cases that were not called due to concerns about low cf‐DNA fetal fraction.^2^, ^46^ Published data on the experience for NIPT for fetal trisomy 13 and trisomy 18 in twin pregnancies is scant but consistent with expectations for singleton pregnancies. As previously noted, there is a special concern for these latter aneuploidies because fetal fraction is often lower than normal.

There are some additional caveats. Most laboratories do not offer cf‐DNA screening for sex chromosome abnormalities or limit the testing to monosomy‐X in multifetal pregnancies. Many sex chromosome abnormalities are mosaic and not confirmed after initially being detected in trophoblasts.^47^ Additionally, results for X‐chromosome aneuploidies need to be interpreted cautiously when using methods that do not distinguish between maternal and fetal genotypes (i.e., counting based NIPT) because maternal‐age related gain and loss of an X‐chromosome in maternal tissues is a recognized cause for false‐positive NIPT results.^48^, ^49^

## 6 VANISHING TWIN PREGNANCIES

The presence of a demised (“vanishing”) twin is common in pregnancies conceived by in vitro fertilization with rates dependent on the number of transferred embryos.^50^, ^51^ A robust estimate of the rate in naturally conceived pregnancies has yet to be determined; one study found, by ultrasound, 54/4746 (1.1%) vanished twin pregnancies in women referred for aneuploidy screening or diagnosis.^52^

A vanishing twin can be identified through cf‐DNA analysis.^53^ Vanishing twins are rarely detected with counting based NIPT if the testing is confined to trisomies 21, 18 and 13 but detection is more common when testing for sex chromosome abnormality is also offered.^54^ The genome‐wide counting‐based approach to NIPT potentially identifies other autosomal trisomies that are common and strongly associated with fetal death and therefore this approach will identify additional vanished twin pregnancies^55^ However, many of these additional abnormalities are also present as confined placental mosaicisms that appear to have little clinical significance.^56^ The SNP‐based NIPT will identify a vanishing dizygotic twin (with or without aneuploidy) provided the maternal plasma contains sufficient fetal cf‐DNA.

Although the clinical utility of routinely detecting vanishing twins through NIPT has not yet been demonstrated, there are individual cases where cf‐DNA analysis may be potentially helpful. This includes identifying situations where a vanished twin rendered serum markers less accurate in an initial screening step^57^; evaluation of women with unexplained vaginal bleeding, spotting or pain^58^; assisting in interpreting inconsistencies between fetal anatomy and cytogenomic results^59^; resolving questionable mosaicism/chimerism^60^; where an initial positive NIPT test using a non‐SNP based method was not confirmed, and in the evaluation of any other pregnancy findings that could be attributable to residual tissue from a vanished twin.

There is conflicting data on the significance of vanishing twin and its impact on pregnancy outcomes.^50^, ^51^, ^58^ In a large prospective study, a vanishing twin was shown to be an independent risk factor for both iatrogenic and spontaneous preterm birth.^52^ Others have also reported these pregnancies to be at risk for growth abnormalities at later gestational ages.^58^ Further studies incorporating data from NIPT may better elucidate these associations.

## 7 OVERVIEW

Although it is impractical to demonstrate efficacy in a large prospective trial, there is substantial indirect evidence to conclude that the use of cf‐DNA testing improves the screening for fetal trisomy in twin pregnancies. NIPT can evaluate zygosity and this can be particularly useful for guidance of pregnancy management in the situation where ultrasound assignment of chorionicity is uncertain or when twin pregnancies are diagnosed later in gestation. Although monozygosity does not imply monochorionicity, dizygotic twins are almost always dichorionic. The need for proper counseling of patients and the importance of an appropriate pregnancy surveillance scheme dictates that the information be made available. The presence of two or more fetal genomes in cf‐DNA, each of which is present in different concentrations, is a challenge in NIPT. While this does lead to higher rates of uninterpretable tests, it is prudent to recognize these situations and consider alternative testing. For some women and their clinicians, identification of a vanished twin through cf‐DNA testing may be useful to adjust obstetrical care. It is therefore reasonable to conclude that NIPT will play an increasingly important role in the clinical services offered to women with multiple pregnancies.

cfDNA testing complements, and does not replace, first trimester ultrasound screening in multiple gestation pregnancies. In addition to providing information about chorionicity in twin pregnancies, first trimester ultrasound identifies abnormal maternal pathology (e.g., Mullerian anomalies, adnexal masses) and fetal abnormalities (e.g., increased nuchal translucency, major structural congenital abnormalities such as anencephaly) which can have a serious impact on outcomes in multifetal gestations. As discussed, NIPT provides superior aneuploidy screening and zygosity information. Together, ultrasound and NIPT facilitate early diagnosis of serious adverse conditions and allows couples to plan the course of the pregnancy or consider options such as pregnancy termination or fetal reduction. Application of these technologies in twin pregnancies provides an excellent illustration of the of the synergy between imaging and laboratory testing.

## CONFLICT OF INTEREST

P. Benn is a consultant and holds stock options in Natera, Inc. He is also on an Advisory Board for Menarini Biomarkers. A. Rebarber is the President of Carnegie Imaging for Women, PLLC & President of Maternal Fetal Medicine Associates, PLLC. The opinions expressed are those of the authors and not based on input from these companies.
